# Tissue-nonspecific Alkaline Phosphatase Regulates Purinergic Transmission in the Central Nervous System During Development and Disease

**DOI:** 10.1016/j.csbj.2014.12.004

**Published:** 2014-12-15

**Authors:** Álvaro Sebastián-Serrano, Laura de Diego-García, Carlos Martínez-Frailes, Jesús Ávila, Herbert Zimmermann, José Luis Millán, María Teresa Miras-Portugal, Miguel Díaz-Hernández

**Affiliations:** a Department of Biochemistry and Molecular Biology, Veterinary School, Complutense University of Madrid, Avda. Puerta de Hierro S/N, 28040 Madrid, Spain; b Instituto de Investigación Sanitaria del Hospital Clínico San Carlos, IdISSC, Madrid, Spain; c Centro de Biología Molecular Severo Ochoa (CSIC-UAM), Madrid, Spain; d Centro de Investigación Biomédica en Red sobre Enfermedades Neurodegenerativas (CIBERNED, ISCIII), Madrid, Spain; e Institute of Cell Biology and Neuroscience, Molecular and Cellular Neurobiology, J. W. Goethe-University, Frankfurt 60438, Germany; f Sanford Children's Health Research Center, Sanford-Burnham Medical Research Institute, La Jolla, CA, United States

**Keywords:** TNAP, Development, Neurodegenerative diseases, Pyridoxal phosphate, ATP, P2X7R

## Abstract

Tissue-nonspecific alkaline phosphatase (TNAP) is one of the four isozymes in humans and mice that have the capacity to hydrolyze phosphate groups from a wide spectrum of physiological substrates. Among these, TNAP degrades substrates implicated in neurotransmission. Transgenic mice lacking TNAP activity display the characteristic skeletal and dental phenotype of infantile hypophosphatasia, as well as spontaneous epileptic seizures and die around 10 days after birth. This physiopathology, linked to the expression pattern of TNAP in the central nervous system (CNS) during embryonic stages, suggests an important role for TNAP in neuronal development and synaptic function, situating it as a good target to be explored for the treatment of neurological diseases. In this review, we will focus mainly on the role that TNAP plays as an ectonucleotidase in CNS regulating the levels of extracellular ATP and consequently purinergic signaling.

## 1 Introduction

Alkaline phosphatases (APs) are ubiquitous ectoenzymes widely distributed in nature from bacteria to humans, suggesting their involvement in important physiological processes. Their main functions consist of catalyzing dephosphorylation and transphosphorylation reactions on a broad spectrum of physiological and non-physiological substrates [1–3]. AP isozymes, encoded by four homologous gene loci, are present in both humans and mice [4–6]. Three of them, known as the placental (PLAP), germ cell (GCAP), and intestinal (IAP) types, are tissue-specific with highly restricted expression, while the fourth isozyme, tissue-nonspecific AP (TNAP), is present in numerous tissues but particularly abundant in mineralizing tissues, the kidneys [7] and the central nervous system (CNS) [8,9]. TNAP is encoded in humans by the ALPL (alkaline phosphatase, liver/bone/kidney) gene and by the Akp2 (alkaline phosphatase 2) gene in mice, both with 12 exons [10–12]. In both species, two different transcripts derived from the same coding region have been described [12–14]. Similar to the rest of the mammalian AP family, TNAP is a homodimeric protein anchored to the cytoplasmic membrane via two GPI moieties [15,16]. Each monomer contains three metallic ions (two zinc molecules and one of magnesium) and one phosphate ion. The central core of each subunit consists of an extended β-sheet flanked by α-helices. Other two identifiable regions are the long N-terminal α-helix and an interfacial flexible loop known as the “crown domain” [2].

TNAP hydrolyzes extracellular inorganic pyrophosphate (PP_i_), a potent mineralization inhibitor, to enable the physiological deposition of hydroxyapatite in bones and teeth [2,17–20]. Hypomorphic mutations in the ALPL gene encoding TNAP lead to accumulation of PP_i_ in the extracellular matrix causing a heritable form of rickets in children or osteomalacia in adults known as hypophosphatasia [6,21–24]. Another substrate of TNAP is pyridoxal-5′-phosphate (PLP, the major active form of vitamin B6) [25]. TNAP converts extracellular PLP into pyridoxal that is taken up into cells and rephosphorylated by intracellular kinases. There it functions as a cofactor for the synthesis of enzymes implicated in the metabolism of several neurotransmitters, such as gamma-aminobutyric acid (GABA) or serotonin [26]. In addition, this enzyme has been described as an ectonucleotidase, being able to cleave all forms of adenosine phosphates influencing purinergic signaling [27]. Along with APs, there are also three major groups of ectonucleotidases: the ecto-nucleoside triphosphate diphosphohydrolases, ecto-5′-nucleotidase, and ecto-nucleotide pyrophosphatase/phosphodiesterases (for an extensive review of the structure and function of the ectonucleotidases see [28]). All these properties of TNAP linked to the fact that it is highly expressed in the brain and the developing spinal cord [8,9,29,30], suggesting a physiological role for TNAP in CNS and its development. Although deficiency in TNAP function leads to seizures, both in patients [25] and in mice [31,32], little is known about the mechanism of action of TNAP in the CNS.

## 2 TNAP Contributes to Early Embryonic Development of the CNS: Proliferation and Migration

During early development of the nervous system, two main processes take place. First, the neural precursors proliferate and generate the characteristic high cellular variability of the brain. Then, cells migrate until they reach their correct position in the brain.

TNAP is strongly expressed in these early stages in the neural tube, and in migrating primordial germ cells [19,29,33], a subpopulation of neuroectodermal cells. In mice, these cells are characterized by moving from the epithelium of the hind gut to the genital ridges around embryonic day 8 (E8) [34]. The high expression of TNAP in these cells during their migration may suggest an unknown role of the enzyme in this process.

Furthermore, strong activity of TNAP has been found around embryonic day 14 (E14) in ventricular and subventricular zones (VZ and SVZ) where neural precursors are placed [35]. Taking into account that these regions are characterized by increased proliferative activity, either during development or in the adult brain [36], it would be reasonable to relate TNAP function with proliferation. Moreover, increasing evidence points to purinergic signaling pathways playing a role in embryonic and adult neurogenesis [35]. The activation of purinergic receptors can transiently increase intracellular Ca^2 +^ concentrations, independently of voltage-dependent Ca^2 +^-channel activation [37], and this increase could be related to cellular proliferation [38]. Studies using embryonic rat slices demonstrated that Ca^2 +^ waves propagating through radial glial cells of VZ are mediated by P2Y1 receptor activation. Disruption of calcium waves induces a reduction of cell proliferation in the VZ [39]. Furthermore purinergic signaling facilitates progenitor cell migration within the developing cortex [17,18]. This suggests that purinergic signal molecules surrounding the precursor cells are essential for proper brain development. We surmise that these progenitor cells employ the ectonucleotidase activity of TNAP to regulate the nucleotide availability in the microenvironment of purinergic receptors [35,40]. In support of this hypothesis, downregulation of TNAP in neural stem cells from adult mice affects differentiation in vitro and possibly also in vivo [41]. The mechanisms underlying the impact of TNAP activity on precursor cell proliferation and possibly migration require further investigation.

## 3 TNAP Regulates Neuronal Differentiation: Axonal Growth

The next essential event during development is axonal growth and guidance. Once the intermediate neurons reach their correct position, axonal elongation towards the proper target is initiated, eventually generating the precise neuronal circuits observed in the mature brain. Guidance cues include neurotrophic factors, neurotransmitters and other signals, both diffusible and associated with the neuronal membrane. These molecules employ attraction and repulsion mechanisms, directing the axonal growth through the control of polymerization and depolymerization of microtubules and microfilaments [42,43].

Around E10.5, strong TNAP activity has been found in cranial nerves and dorsal roots that may be related to pioneer growth cones [29]. One hypothesis for the involvement of this enzyme in axonal elongation might relate to the ability of TNAP to interact with extracellular matrix proteins, such as collagens, through its loop region [44]. Another hypothesis might relate to the involvement of TNAP in the metabolism of extracellular nucleotides modulating purinergic signaling [27]. In the nervous system, purines act as neurotransmitters mediating not only rapid effects, but also trophic effects inducing changes in metabolism, structure and function [45]. For example, well-established models of axonal growth using neural explants of rat embryos at E12 demonstrate that ATP is able to reduce motoneuron neurite extension [46]. ATP behaves as a neurotransmitter in the CNS activating both, ionotropic P2X receptors (P2XRs) [47–49] and metabotropic P2Y receptors [50]. Activation of ionotropic P2XRs induces transient increases in cytosolic Ca^2 +^ concentrations [37] including in growth cones [51]. This increase negatively regulates the rate of axonal outgrowth. To the contrary, reduction of intracellular Ca^2 +^ levels in axonal growth cones accelerates axonal elongation [52]. Moreover, in vitro studies using cultured hippocampal neurons have shown that ATP can induce an intracellular Ca^2 +^ increase in the axonal growth cone, generating a Ca^2 +^ wave mainly at the distal region of the axon [51] where P2XRs are localized. This focal Ca^2 +^ influx correlates with changes in growth cone morphology, from lamellipodial to filopodial extensions. Pharmacological and molecular biology tools have identified the P2X7R as the ATP receptor inducing these changes. Interestingly, specific P2X7R antagonists induce a significant increase in axonal length [51].

**Fig. 1:**
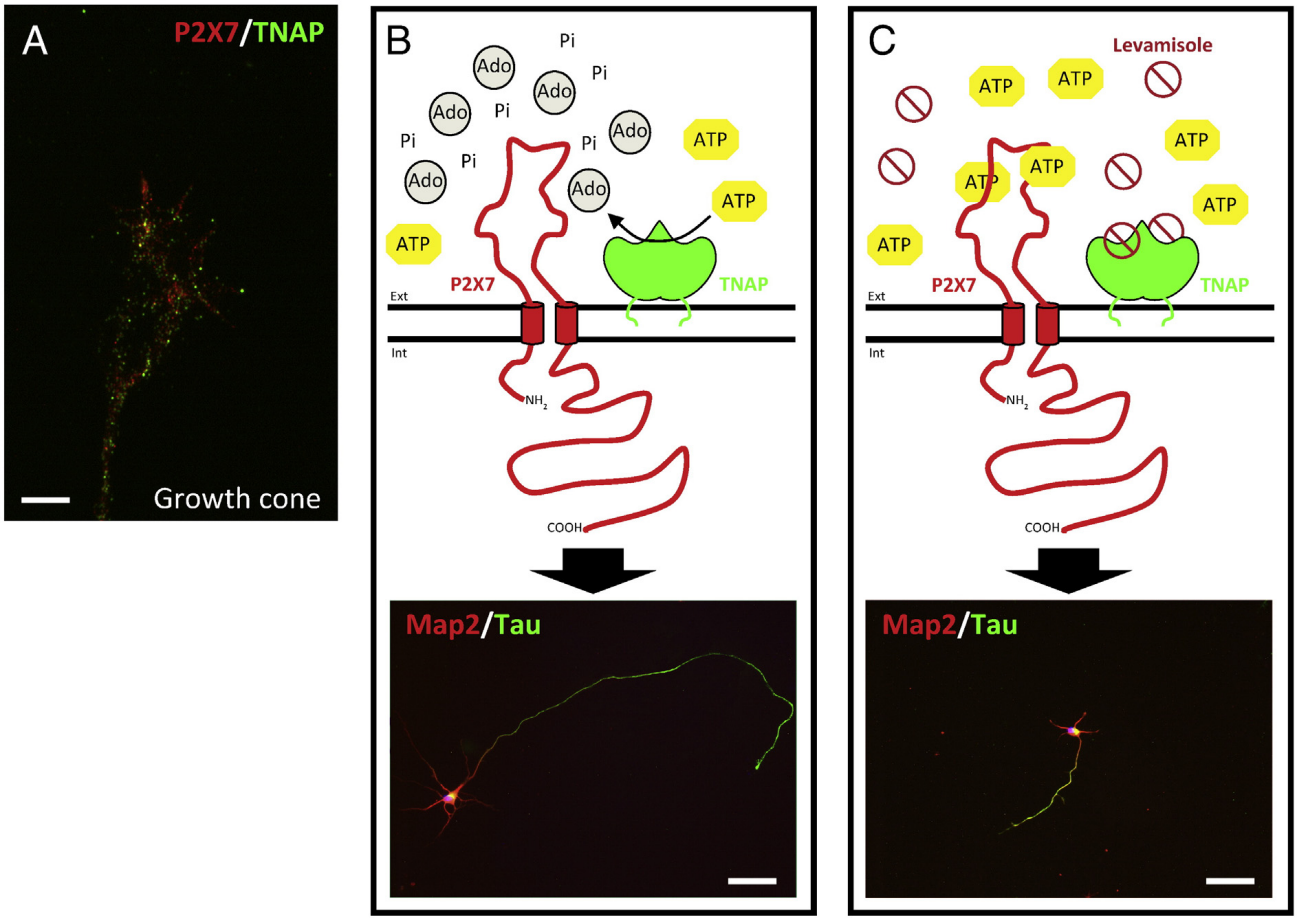
Schematic representation of the axonal growth regulation by the coordinated action of TNAP and P2X7R. A) Immunofluorescence image of the axonal growth of hippocampal neurons fixed at 3 DIV and stained with antibodies against TNAP (green) and P2X7R (red). The image shows the presence of both proteins at the growth cone of hippocampal neurons. Scale bar: 20 μm. B) TNAP hydrolyzes the physiological agonist of P2X7R, ATP, in the proximal environment of the receptor, which negatively regulates the activation of this receptor favoring in this way the axonal growth. C) The pharmacological inhibition of TNAP by levamisole produces an increase of ATP in the proximal environment of P2X7R, event that favor the activation of the receptor and then decreasing the axonal growth. The immunofluorescence images of lower panels show hippocampal neurons (3 DIV) stained with antibodies against axonal molecular markers, Map2 (red) and Tau (green), under normal condition (B) or treated with TNAP antagonist, levamisole, inhibiting axonal growth (C). Scale bar, B and C: 50 μm. Ext: extracellular space. Int: intracellular space. Ado: adenosine. Pi: inorganic phosphate. (For interpretation of the references to color in this figure legend, the reader is referred to the web version of this article.)

Additional studies, again with cultured hippocampal neurons, showed that during the first days of culturing extracellular ATP levels are considerably reduced [53]. The reduced levels of extracellular ATP correlate with a significant increase in TNAP activity, especially at the axonal growth cone. It is important to note that during the three initial days in culture, one of the neurites emerges from the cell body to become the axon. Pharmacological inhibition of TNAP maintains high levels of extracellular ATP in the culture media and inhibits the growth and branching of axons (Fig. 1B and C) [53]. Neither activation nor inhibition of adenosine receptors influenced axonal growth, excluding the contribution of adenosine in this process, the principal product generated by extracellular hydrolysis of ATP by TNAP [53]. The presence of TNAP at growth cones suggests a close functional interrelation between P2X7R and the ectonucleotidase (Fig. 1A). TNAP probably induces axonal elongation by hydrolyzing ATP in the immediate environment of the receptors, thus preventing the activation of P2X7R. Of note, inhibition of P2X7R reduced TNAP expression while addition of exogenous TNAP enhanced P2X7R expression revealing a novel relationship between both proteins at the transcriptional level [53].

## 4 TNAP and Synaptic Function

Once the axon reaches the proper target, it has to establish synaptic contacts. TNAP is selectively expressed in the synaptic cleft of sensory cortical areas in adult primates [8] and in humans [54]. As shown by deprivation paradigms in monkeys TNAP activity is regulated by sensory experience [8]. It is of interest that the high activity of TNAP in the cortex coincides with the peak of synaptogenesis [55,56], suggesting a functional involvement of TNAP in synapse formation and maturation. Recent studies demonstrated the presence of TNAP in the retina of several vertebral species, including humans, suggesting a role in retinal neurotransmission [57]. While the precise biological functions of TNAP at these CNS sites remain to be further elucidated, emerging evidence suggests that TNAP may act through metabolic pathways additional to hydrolyzing extracellular nucleotides. The levels of PLP are regulated by TNAP [58]. PLP is a co-factor of glutamic acid decarboxylase (GAD65) [59] essential for GABA synthesis. GABA is one of the main inhibitory neurotransmitters in the CNS. Changes in its concentration may induce an imbalance between excitatory and inhibitory synaptic responses. In addition, PLP is necessary for the synthesis of serotonin, dopamine, epinephrine and norepinephrine [26,60].

As noted above, TNAP is potentially involved in purinergic transmission by producing nucleoside receptor substrates through the extracellular hydrolysis of ATP to adenosine [28]. Adenosine has been widely described as a neuromodulator in the CNS, and the activation of its receptors is implicated in several physiological processes [61]. Further to this, studies using nerve endings from the rat midbrain as a model demonstrated that the adenosine generated by TNAP, is sufficient to activate presynaptic A1 adenosine receptors. This in turn increased the affinity and response of presynaptic ionotropic nucleotide and dinucleotide receptors at the same nerve endings [62,63]. Additionally, several neurotransmitters like acetylcholine (ACh), glutamate or GABA could be released by vesicle-dependent mechanisms when these ionotropic nucleotide receptors were activated [64–66]. This suggests that TNAP might modulate synaptic function by regulating the availability of ligands at nucleotide and nucleoside receptors.

TNAP is often co-expressed with other ectonucleotidases [27] which could redundantly generate adenosine from extracellular nucleotides in different regions of the nervous system, including the somatosensory system or the hippocampus. In both cases, the adenosine generated acts by inhibiting dorsal root ganglion or spinal neurons and hippocampal activity, respectively [67,68].

Finally, pioneering studies using TNAP knockout mice analyzed the consequences of the lack of TNAP on postnatal brain development, specifically from the first to the tenth day of postnatal development. During these days, at the spinal cord level, the relative amount of white matter suffers a considerable decrease in the knockout mice when compared to their control littermate wild-type mice. This fact was accompanied by a decrease in the g-ratio (axon diameter/fiber diameter) of the myelinated fibers and in the thinning of the myelin sheath [32,69]. In the cerebral cortex, myelinated axons, found to be present in wild-type littermate mice, were absent in TNAP knockout animals at seventh and eighth postnatal days. Remarkably, the cerebral cortex from these mutant mice contains a high proportion of immature synapses supporting the hypothesis that TNAP plays also a role in synaptogenesis [69].

## 5 Implication of TNAP in Neurodegenerative Diseases: Alzheimer's Disease and Epilepsy

As mentioned above, TNAP hydrolyzes a wide spectrum of monoesters of phosphoric acid. These properties together with the abnormalities in myelination and synaptogenesis observed in the TNAP knockout mice, a model of infantile hypophosphatasia, implicate an important role of this enzyme in neuronal development [32,69]. Therefore, TNAP emerges as a plausible target for the treatment of neurological diseases where the synaptic function is altered such as epilepsy or Alzheimer's disease.

### 5.1 Alzheimer's Disease (AD)

In 2006 the incidence of AD was around 30 million cases throughout the world [70], but this figure has been increasing each year since then. At histopathological levels, this disease is characterized by the presence of two aberrant structures: extracellular senile plaques composed by amyloid beta peptide, and intracellular neurofibrillary tangles (NFTs) mainly formed by hyperphosphorylated tau protein [71].

Hyperphosphorylated tau initially appears in the entorhinal cortex and spreads from there to surrounding regions like the hippocampus [72]. In these brain regions it has been found that a relationship between the extent of tangles and neuronal death exists [73–75]. Regarding the underlying mechanism, it has been recently reported that tau protein induces a toxic effect through the activation of muscarinic receptors, specifically M1 and/or M3 receptors [76,77]. But, why tau and not other muscarinic agonists like ACh, is able to induce this neurotoxic effect? We can find the answer in three differential facts; first tau has around one order of magnitude higher affinity for muscarinic receptors than ACh. Second, a repeated stimulation of the muscarinic receptor by ACh induced receptor desensitization, and this phenomenon failed when they were stimulated by tau. And finally, tau protein is very stable in the interstitial space, remaining intact in this location more time than ACh [78]. All these data can explain, at least in part, the toxic effect associated with tau protein in AD.

**Fig. 2:**
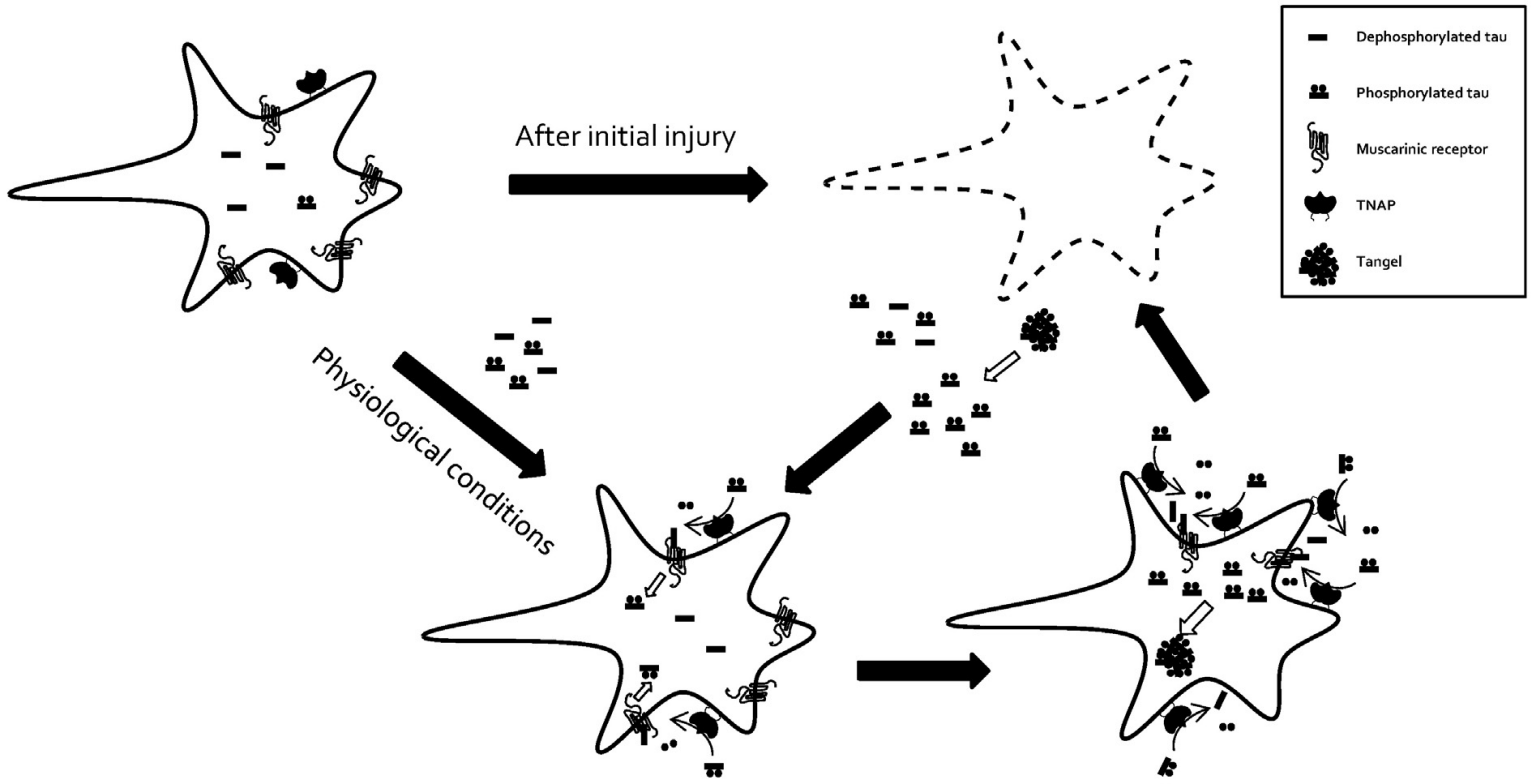
Schematic representation illustrating the involvement of TNAP in the progression of Alzheimer's disease. First, tau protein reaches the interstitial space as a consequence of an initial undetermined injury or by its own vesicular release under physiological conditions. This tau protein is dephosphorylated by TNAP becoming an active ligand of muscarinic receptors. The activation of muscarinic receptors by tau has three main consequences; an increase of intracellular level of calcium and hyperphosphorylated tau, and an increase of TNAP levels. Subsequently of these effects, a positive feedback loop is generated in which the final consequence is cell death. With the rupture of the plasma membrane after the cell death, the intracellular contents are released to the interstitial space, increasing the extracellular levels of hyperphosphorylated tau. The NFTs (intracellular neurofibrillary tangles) suffer slow disassembly and degradation, allowing the proteins to reach distant brain regions that results in spreading of this neurodegenerative process.

It has been recently reported that extracellular hyperphosphorylated tau protein coming from damaged neurons must be dephosphorylated to become an agonist of a muscarinic receptor and induce the unbalances of the intracellular calcium homeostasis that finally triggers neuronal death [79]. Furthermore, this activation by dephosphorylated tau also increases TNAP expression and the phosphorylation levels of intracellular tau [76,79]. Taking all these data together, we can postulate the next scenery that is summarized in Fig. 2. Briefly, after an initial neuronal damage or maybe by its own vesicular release [80] hyperphosphorylated tau reaches the extracellular space where it is dephosphorylated by TNAP. Afterwards, dephosphorylated tau can activate muscarinic receptors producing an increase in the intracellular levels of calcium, phosphorylation of intracellular tau protein and the expression of TNAP. This last event will cause a more efficient dephosphorylation of the extracellular hyperphosphorylated tau. The final result is a positive feedback mechanism that maintains constant muscarinic receptor activation that could provoke the neuronal death by the imbalance of intracellular calcium homeostasis. But in addition, this mechanism also generates the formation of new intracellular tangles, prior to the cell death. With the rupture of the plasma membrane after the cell death, the intracellular contents are released to the interstitial space, increasing the extracellular levels of hyperphosphorylated tau. The NFTs suffer slow disassembly and degradation, allowing the proteins to reach distant brain regions which results in spreading of this neurodegenerative process [79].

Supporting this hypothesis, preclinical assays tested on more than 100 AD patients have shown that TNAP activity is significantly increased in the hippocampus of AD patients compared with age-related controls, independent of whether they were diagnosed as sporadic or genetic AD. Interestingly, this study also demonstrated an increase of TNAP levels in the plasma of the AD patients [81], suggesting that TNAP is a good biomarker of disease progression.

### 5.2 Epilepsy

Epilepsy is a common and chronic group of neurological disorders characterized by recurrent unprovoked seizures, which range from brief and practically undetectable, to longer periods of violent convulsions. It affects about 50 million people worldwide [82]. Epileptic seizures are the result of excessive and abnormal hypersynchronous firing of neurons in the brain [83].

Bearing in mind that TNAP regulates the availability of PLP [25], the cofactor implicated in GABA synthesis [26], the first studies directed to elucidate the causes of the spontaneous epileptic seizures observed in TNAP knockout mice [31,32] focused on the dysregulation of GABAergic signaling, responsible for inhibition of neuronal activity. Administration of vitamin B6 (pyridoxal) was found to suppress these seizures [84] and the authors suggested that the epileptic seizures observed in TNAP knockout mice result from GAD dysfunction resulting from reduced hydrolysis of extracellular PLP and subsequent shortage of intracellular PLP. This could also explain the abnormal morphology of the lumbar nerve roots and myelination defects observed in these mice [32,69]. Finally, the role of TNAP as an ectonucleotidase must be further investigated. Considering that P2XRs have been widely related to epilepsy [85] as well as TNAP is able to regulate ligand availability (ATP) in the environment of P2XRs, together with recently reported work where it is described that PLP can antagonize the response induced by the activation of some P2XRs [86], we can suggest that the contribution of TNAP to the seizures suffered by TNAP null mice may be due to multiple factors. However, to validate this hypothesis deeper and exhaustive studies should be done.

## 6 Concluding Remarks

The studies presented here demonstrate that under normal conditions TNAP plays a key role during CNS development, being involved on neural differentiation as well as in the establishment and maintenance of the synaptic contact. However, alterations on its normal function have been associated with some neurological diseases, such as epilepsy or AD. Considering that the role of TNAP in the CNS is just starting to be elucidated, new studies have to be performed to identify the factors that are altering its normal function on these diseases. On the other hand, due to the wide distribution that TNAP presents in the whole body, new specific antagonists of TNAP with a restricted distribution to CNS should be developed. In addition, according to what was mentioned in Section 5.2, to consider this enzyme as a validated therapeutic target to treat AD, the combination of these new compounds with selective P2X antagonists would avoid epileptic seizures derived from the maintained inhibition of TNAP.
